# Post-Synthetic and In Situ Vacancy Repairing of Iron Hexacyanoferrate Toward Highly Stable Cathodes for Sodium-Ion Batteries

**DOI:** 10.1007/s40820-021-00742-z

**Published:** 2021-12-03

**Authors:** Min Wan, Rui Zeng, Jingtao Meng, Zexiao Cheng, Weilun Chen, Jiayu Peng, Wuxing Zhang, Yunhui Huang

**Affiliations:** 1 School of Mechanical Science and Engineering, Huazhong University of Science and Technology, Wuhan, 430074 People’s Republic of China; 2 School of Chemistry and Environmental Engineering, Wuhan Polytechnic University, Wuhan, 430023 People’s Republic of China; 3 State Key Laboratory of Materials Processing and Die & Mould Technology, School of Materials Science and Engineering, Huazhong University of Science and Technology, Wuhan, 430074 People’s Republic of China

**Keywords:** Iron hexacyanoferrate, Cathode, Vacancy repairing, Sodium-ion batteries

## Abstract

Iron hexacyanoferrate (FeHCF) is a promising cathode material for sodium-ion batteries. However, FeHCF always suffers from a poor cycling stability, which is closely related to the abundant vacancy defects in its framework. Herein, post-synthetic and in-situ vacancy repairing strategies are proposed for the synthesis of high-quality FeHCF in a highly concentrated Na_4_Fe(CN)_6_ solution. Both the post-synthetic and in-situ vacancy repaired FeHCF products (FeHCF-P and FeHCF-I) show the significant decrease in the number of vacancy defects and the reinforced structure, which can suppress the side reactions and activate the capacity from low-spin Fe in FeHCF. In particular, FeHCF-P delivers a reversible discharge capacity of 131 mAh g^−1^ at 1 C and remains 109 mAh g^−1^ after 500 cycles, with a capacity retention of 83%. FeHCF-I can deliver a high discharge capacity of 158.5 mAh g^−1^ at 1 C. Even at 10 C, the FeHCF-I electrode still maintains a discharge specific capacity of 103 mAh g^−1^ and retains 75% after 800 cycles. This work provides a new vacancy repairing strategy for the solution synthesis of high-quality FeHCF.

**Supplementary Information:**

The online version contains supplementary material available at 10.1007/s40820-021-00742-z.

## Highlights

- Post-synthetic and in-situ vacancy repairing strategies effectively decrease the defects in FeHCF.
- Vacancy reduction improves the structure and cycling stability of FeHCF.
- Vacancy reduction boosts the capacity contribution from low-spin Fe in FeHCF.

**Supplementary Information:**

The online version contains supplementary material available at 10.1007/s40820-021-00742-z.

## Graphical abstract

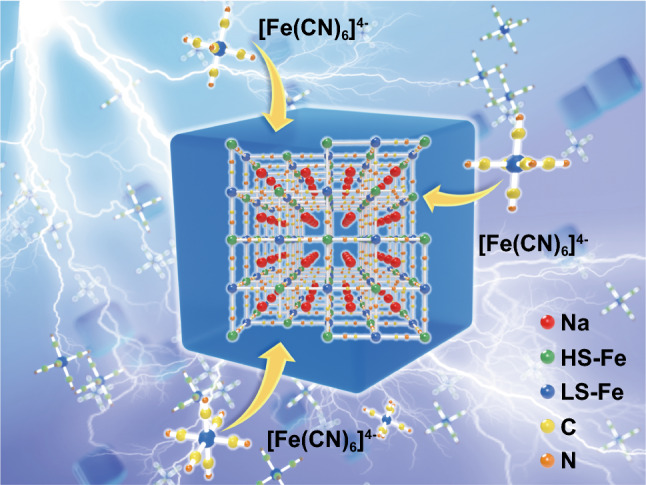

## Introduction

Recently, sodium-ion batteries (SIBs) have been considered as a promising energy storage technology for large-scale power grid energy storage due to the high natural abundance and low cost of sodium [1–3]. The performance of SIBs is strongly affected by the cathode materials, which are the Na^+^ providers in SIBs. Generally, three types of cathode materials have widely been investigated for SIBs: layered transition metal oxides, polyanionic compounds, and Prussian blue analogues (PBAs) [4, 5]. Among them, PBAs have attracted much attention because of their low cost, facile synthesis, and high theoretical specific capacity [6, 7]

PBAs generally have the chemical formula Na_*x*_M_1_[M_2_(CN)_6_]_*y*_□_*1-y*_·*n*H_2_O, where 0 < x < 2, 0 < y < 1, M stands for transition metal ions, and □ is the vacancy of [M_2_(CN)_6_] which is usually occupied by coordinated H_2_O [8]. When both M_1_ and M_2_ are Fe, the resulting iron hexacyanoferrate (FeHCF) could provide a theoretical specific capacity of ~ 170 mAh g^−1^. Nevertheless, large amounts of Fe(CN)_6_ vacancies can be formed in the solution synthesis of FeHCF owing to the rapid precipitation process. These lattice defects with coordinated water can not only break the structural integrity and reduce the active Na^+^ storage sites, but can also cause a structural collapse during the charge–discharge process, leading to deterioration of the electrochemical performance [9–13]. To overcome this issue, most research focuses on reducing the crystallization rate, resulting in a lower vacancy content in the framework [14–16]. Guo and Goodenough prepared FeHCF cathodes with low vacancy defects via the hydrothermal method using a single iron source, and the high-quality FeHCF exhibited a high specific capacity and excellent cycling stability and rate performance [17, 18]. Moreover, a co-precipitation method with two iron sources and chelating agents was also developed to prepare FeHCF with low defects. Our group explored the chelating effects of sodium citrate and synthesized high-quality Na_1.70_FeFe(CN)_6_, which exhibits a discharge specific capacity of approximately 120 mAh g^−1^ at 200 mA g^−1^ [19]. Recently, Cao et al. reported a low-defect and Na-rich FeHCF using trisodium citrate and NaCl as additive agents. The obtained Na_1.81_Fe[Fe(CN)_6_]_0.83_·2.04 H_2_O electrode can provide a specific capacity of 77 mAh g^−1^ at a current density of 1400 mA g^−1^ (10 C) [20]. Chou et al. systematically investigated factors such as chelating agents, dropwise addition, and Ar protection. After optimizing the synthetic conditions, Na-rich Na_1.76_FeFe(CN)_6_ with a high crystallinity and microparticles can deliver a specific capacity of approximately 115 mAh g^−1^ with a high capacity retention (76% after 1000 cycles at 200 mA g^−1^) [21]. Until now, the removal of vacancies in FeHCF remains a significant challenge due to the low productivity of the above strategy [22].

Alternatively, it has previously been reported that the vacancy defects in PBAs can be repaired via solid reactions. Liao et al. showed that NaFeFe(CN)_6_ with a low water content can be synthesized by the solid-state reaction between Fe[Fe(CN)_6_]_3/4_□_1/4_ and Na_4_Fe(CN)_6_, whereby the obtained NaFeFe(CN)_6_ electrode exhibits an excellent cycling stability [23]. Goodwin et al. further demonstrated the mechanochemical vacancy filling in Mn[Co(CN)_6_]_2/3_□_1/3_ via K_3_Co(CN)_6_ [24]. Therefore, post-synthetic control over vacancy fractions in PBAs can be achieved using a mechanochemical approach.

Inspired by the above mechanochemical approach to repair the vacancy defects in PBAs, this work proposes post-synthetic and in-situ vacancy repairing strategies for the solution synthesis of FeHCF. Based on DFT calculations, the vacancy repairing mechanism is established and its effect on the sodium storage performance is discussed. Both post-synthetic and in-situ vacancy repaired FeHCF products show excellent electrochemical performance because of the significantly decreased vacancy defects and the reinforced structure. Thus, our work sheds new light on the control over vacancy fractions in PBAs.

## Experimental Section

### Materials

#### *Post Vacancies Repairing of FeHCF Microcubes* (FeHCF-P)

All the reagents were purchased from Sigma Aldrich and Sinopharm Group. Based on our former work [25], FeHCF microcubes can be prepared by a co-precipitation method. Typically, 1.112 g FeSO_4_·7H_2_O and 20 g sodium citrate were dissolved in 100 mL deionized water to form solution A, and 0.484 g Na_4_Fe(CN)_6_·10H_2_O was dissolved into 100 mL deionized water to form solution B. The above two precursor solutions were then mixed directly with vigorous stirring for 1 h and then aged at 25 ℃ for 12 h. The precipitates were collected by centrifugation, then washed thoroughly with distilled water and alcohol, and finally dried in a vacuum oven at 120 ℃ for 24 h. The sample collected is denoted as FeHCF. As for post vacancies repairing, 40 g Na_4_Fe(CN)_6_·10H_2_O was dissolved into 100 mL deionized water at 40 ℃, followed by the addition of 0.5 g FeHCF microcubes. The above suspension was stirred at 40 ℃ for 24 h. The precipitates were collected by centrifugation, then washed thoroughly with distilled water and alcohol, and finally dried in a vacuum oven at 120 ℃ for 24 h. The sample collected is denoted as FeHCF-P.

#### *In-situ Vacancies Repairing of FeHCF Nanocubes* (FeHCF-I)

Typically, 0.556 g FeSO_4_·7H_2_O and 5 g sodium citrate were dissolved in 100 mL deionized water, and 40 g Na_4_Fe(CN)_6_·10H_2_O was dissolved into 100 mL deionized water at 40 ℃. The above two precursor solutions were then mixed with stirring and maintained at 25 ℃ for 24 h. The precipitates were collected by centrifugation, then washed thoroughly with distilled water and alcohol, and finally dried in a vacuum oven at 120 ℃ for 24 h. The sample collected is denoted as FeHCF-I.

### Characterization

The crystalline phases were characterized with X-ray powder diffraction (XRD, Panalytical X’pert PRO MRD, Holland) equipped with Cu Kα radiation. JSM 7600F field emission scanning electron microscope (FE-SEM) was used to observe the morphology. Transmission electron microscopy (TEM) and high-resolution TEM (HR-TEM) observations were carried out with JEM-2100 electron microscope. Thermogravimetry (TG) measurement was performed on a Netzsch STA 449F3 analyzer from 30 to 500 °C at a rate of 10 °C min^−1^ in N_2_. The chemical composition was determined by the direct-reading inductively coupled plasma emission spectrometer (ICP-OES, Optima 4300DV, USA) for Na, Fe contents, and by elemental analysis (EA, Vario Micro cube, German) for C, N contents. X-ray photoelectron spectroscopy (XPS) was used to analyze the valence state of elements by Shimadzu/Kratos Axis Ultra-DLD with Al Kα. Nitrogen adsorption/desorption isotherms were conducted on a Micromeritics analyzer (ASAP 2020) at 77 K. The vibration states of existing functional groups were examined via Fourier transform infrared spectroscopy (FTIR, Thermo Nicolet Nexus 670 FTIR spectrometer).

### Electrochemical Measurement

The tests were conducted in 2032 type coin cells, which were assembled in an argon-filled glove box with water and oxygen content less than 0.1 ppm. Sodium foil was employed as the counter electrode. The working electrode consisted of 70 wt active materials, 10 wt.% Ketjen black carbon, 10 wt% Super P, and 10 wt% polyvinylidene fluoride (PVDF) binder. The active mass of cathode is about 1.5 mg cm^−2^. Na-ion coin cells were assembled by using the above working electrode, counter electrode, glass fiber from Whatman as the separator, and 1 mol L^–1^ NaClO_4_ in a mixed solvent of ethylene carbonate (EC) and diethyl carbonate (DEC) (vol/vol = 1:1) with 5 vol% fluoroethylene carbonate (FEC) as electrolyte. The electrolyte amount is about 150 μL in each coin cell. Cyclic voltammetry (CV) and chronopotentiometry measurements were carried out on an electrochemical workstation (CHI660e, ChenHua Instruments Co.) at a scan rate of 0.2 mV s^−1^. The charge/discharge tests were conducted on a testing system (Land Electronics, China) within a voltage window of 2.0 – 4.2 V. All electrochemical tests were conducted at 25 °C. As for the dissolution tests, the coin cells were assembled by FeHCF, FeHCF-P, and FeHCF-I electrodes with the same mass, and electrolyte with the same volume. After 300 cycles at 1C, ICP analysis was conducted to detect the Fe concentration in the electrolyte for FeHCF-P.

### Computational Methods

Density functional theory (DFT) calculations were carried out to study the interactions between FeHCF and water, OH^−^ and [Fe(CN)_6_]^4−^. All calculations were performed using the Materials Studio 2019 CASTEP program from Accelrys [26]. The Perdew-Burke-Ernzerhof (PBE) generalized gradient approximation (GGA) was utilized for the exchange–correlation energy [27, 28]. The core electrons were treated by an ultrasoft pseudopotential. The minimization algorithm used was the Broyden–Fletcher–Goldfarb–Shanno (BFGS) scheme [29]. The valence electron functions were expanded into a set of numerical atomic orbitals using a double-numerical basis with polarization functions (DNP). The unit cell of the FeHCF surface was applied and a vacuum region of 20 Å was used. The reciprocal space was sampled with a (1 × 1 × 1) k-point grid that was automatically generated using the Monkhorst − Pack method. The cut-off energy was 500 eV. To obtain accurate results, the atomic coordinates were optimized by minimizing the total energy and atomic forces. The convergence criteria were as follows: the maximal force on the atoms was 0.03 eV Å^−1^, the stress on the atomic nuclei was less than 0.05 GPa, the maximal atomic displacement was 0.001 Å, and the maximal energy change per atom was 1.0 × 10^−5^ eV. For the interaction resultant system, the total energy was calculated at a single point.

The binding energy between FeHCF and water (△*E*_FeHCF-water_) can be expressed as:

1 $$\vartriangle E_{{{\text{FeHCF}} - {\text{water}}}} = E_{{{\text{FeHCF}} - {\text{water}}}} {-}E_{{{\text{FeHCF}}}} {-}E_{{{\text{water}}}} ,$$

where *E*_FeHCF-water_, *E*_FeHCF_ and *E*_water_ refer to the single-point energy of the PB-water system, FeHCF, and water, respectively. The binding energies between FeHCF and OH^−^, and between the PB and [Fe(CN)_6_]^4−^ were also calculated in a similar way. The binding energy between FeHCF and Na^+^ (△*E*_FeHCF-Na_) can be expressed as:

2 $$\vartriangle E_{{{\text{FeHCF}} - {\text{Na}}}} = E_{{{\text{FeHCF}} - {\text{Na}}}} {-}E_{{{\text{FeHCF}}}} {-}E_{{{\text{Na}}}} ,$$

where *E*_FeHCF-Na_, *E*_FeHCF_, and *E*_Na_ represent the single-point energy of the FeHCF-Na^+^ system, FeHCF, and the Na^+^ ion, respectively.

## Results and Discussion

### Post-synthetic Vacancy Repairing Strategy

**Fig. 1:**
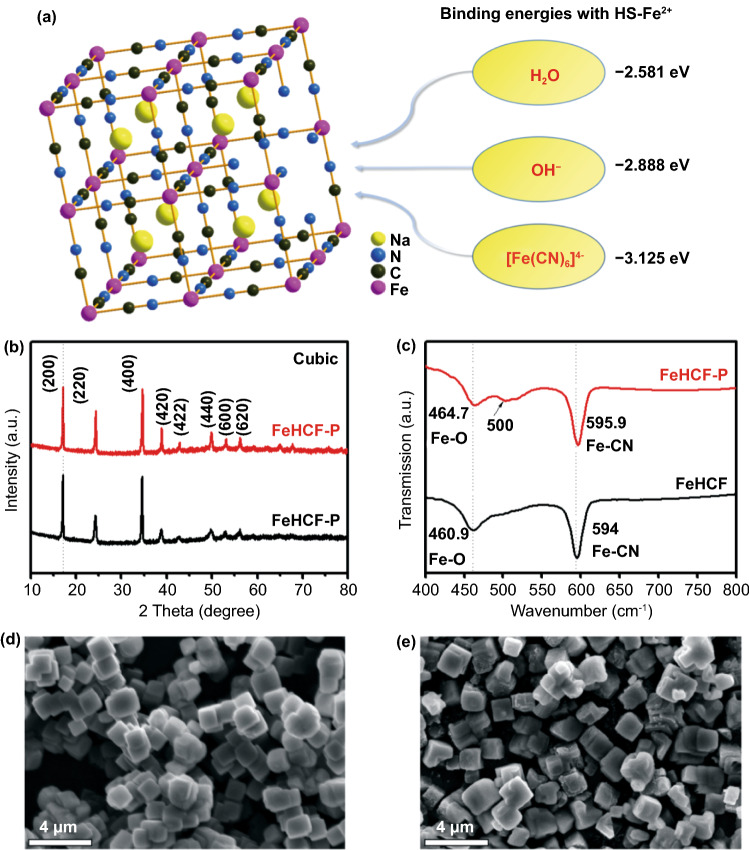
**a** Binding energies between HS-Fe^2+^ in FeHCF with different ligands. **b** XRD patterns and **c** FTIR spectra of FeHCF and FeHCF-P. SEM images of **d** FeHCF and **e** FeHCF-P

In the solution synthesis of FeHCF, high spin Fe^2+^ (HS-Fe^2+^) cations in the vacancies are exposed to H_2_O, OH^−^, and [Fe(CN)_6_]^4−^ anions. DFT calculations show that the binding energy between HS-Fe^2+^ and [Fe(CN)_6_]^4−^ is the lowest, which indicates that HS-Fe^2+^ cations in the vacancies are more likely to coordinate with the [Fe(CN)_6_]^4−^ anions (Fig. 1a). However, in previous reports [16, 19, 25], the vacancies have always been filled by the coordinated water because the concentration of the [Fe(CN)_6_]^4−^ anions is generally quite low in the precursor solution (< 0.01 mol L^−1^). Thus, in order to repair the vacancies in FeHCF, a highly concentrated [Fe(CN)_6_]^4−^ solution is necessary.

Based on the above analysis, a post-synthetic vacancy repairing strategy is proposed by adding the vacancy-rich FeHCF to a 0.8 mol L^−1^ Na_4_Fe(CN)_6_ solution. According to elemental analysis and thermogravimetric analysis (TGA) results (Table S1), the chemical formulas of the FeHCF and FeHCF-P (post-synthetic vacancy repaired FeHCF) samples were calculated as Na_1.66_Fe[Fe(CN)_6_]_0.81_□_0.19_·2.32H_2_O and Na_1.53_Fe[Fe(CN)_6_]_0.86_□_0.14_·2.40H_2_O, respectively. The results demonstrate that the quantity of vacancy defects in FeHCF is significantly reduced (by ~ 26%) after post-synthetic vacancy repairing. The slight decrease in the Na content can be ascribed to the oxidation of Fe^2+^ during the repairing process, which is consistent with the XPS data (Fig. S1). The XRD patterns show that the post-synthetic vacancy repaired FeHCF-P has the same cubic structure as FeHCF (JCPDS No. 01–0239, Fig. 1b). However, the Fourier transform infrared (FTIR) spectra show that the absorption peak of FeHCF-P at 464.7 cm^−1^, corresponding to the Fe–O bonds, is weakened after vacancy repairing [6], and a new absorption peak appears at ~ 500 cm^−1^, which can be assigned to the Fe-CN-Fe bonds [30], indicating that the quantity of Fe–O bonds is reduced on the surface (Fig. 1c). In addition, the absorption peak of the Fe-CN bond shifts from 594 cm^−1^ for FeHCF to 595.9 cm^−1^ for FeHCF-P, indicating strengthened Fe-CN bonds for FeHCF-P [31, 32], therefore, a more robust framework of FeHCF-P as a result of vacancy repairing. The SEM images show that both FeHCF and FeHCF-P exhibit a regular cubic structure with an average particle size of ~ 2 μm (Fig. 1d, e). However, the cubic shape of FeHCF-P was slightly damaged during the repairing process due to the unstable structure in FeHCF, which accordingly resulted in a slight increase in the specific surface area, from 8.28 to 12.83 m^2^ g^−1^ (Fig. S2). The TGA results indicate that the weight loss of FeHCF-P (26%) was clearly lower than that of FeHCF (35%) when the temperature escalated to 500 ℃, confirming that FeHCF-P achieves a better thermal stability owing to its robust framework after vacancy repairing (Fig. S3) [33].

**Fig. 2:**
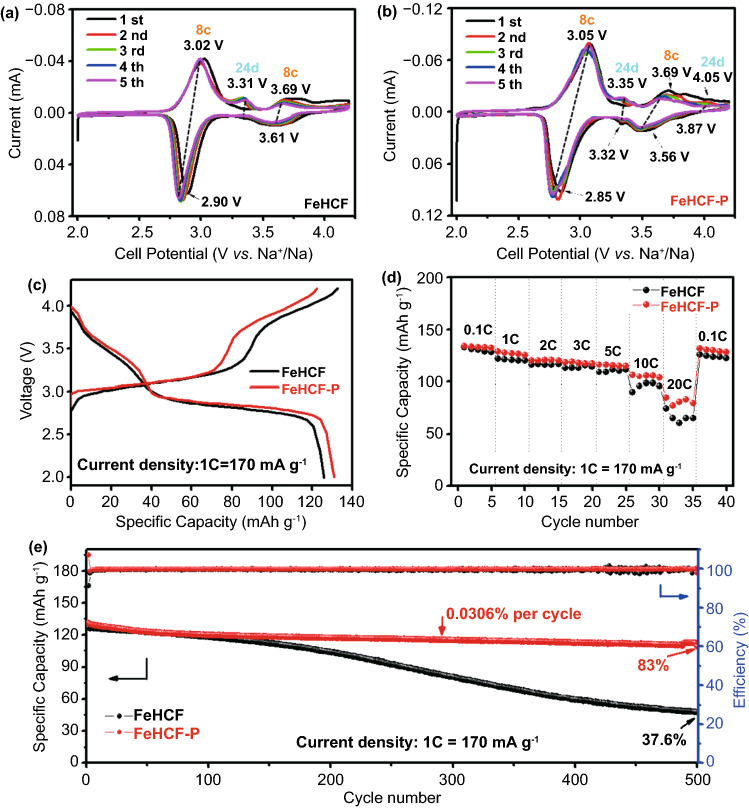
Cyclic voltammetry profiles of **a** FeHCF and **b** FeHCF-P at a scanning speed of 0.2 mV s^−1^. **c** Charge–discharge curves at 1 C. **d** Rate performance and **e** cycling performance under 1 C of FeHCF and FeHCF-P samples

In the CV curves, different redox peaks are observed due to the redox reactions related to Na^+^ ions at various location sites of the framework (Fig. 2a, b). According to our previous theoretical calculations, the most stable Na^+^ sites are located at 8c (body-center) and 24d (face-center) in a defect-free and water-free framework [19]. Therefore, four redox reactions should exist for HS-Fe and LS-Fe (low-spin Fe^2+^) accompanied by the insertion/extraction of Na^+^ at the 8c and 24d sites, i.e., Fe_HS_-8c, Fe_HS_-24d, Fe_LS_-8c, and Fe_LS_-24d, respectively. However, the redox couple Fe_LS_-24d is not observed in the CV curve of FeHCF. After post-synthetic vacancy repairing, a weak oxidation peak appears at 4.05 V corresponding to Fe_LS_-24d and the reduction peak (3.32 V) of Fe_HS_-24d becomes more prominent, confirming that vacancy repairing can effectively enhance the Na^+^ storage capability at 24d sites. Figure 2c shows their initial charge and discharge curves at a current density of 1C (1 C = 170 mA g^−1^). FeHCF-P delivers a reversible discharge capacity of 131 mAh g^−1^, which is higher than that of FeHCF (125.8 mAh g^−1^), indicating that the reduction of vacancy defects could increase the Na^+^ storage sites. Figure 2d shows the rate performances of FeHCF and FeHCF-P electrodes at different current densities. The FeHCF-P electrode exhibits better electrochemical kinetics than FeHCF, and delivers the discharge capacities of 121 mAh g^−1^ at 2 C and 115 mAh g^−1^ at 5 C. Even at the high rate of 20 C, it still retains a discharge capacity of 75 mAh g^−1^. The excellent rate performance of FeHCF-P can be ascribed to the decreased charge transfer resistance after vacancy repairing (Fig. S4). The cycling performance at 1 C is shown in Fig. 2e. The FeHCF-P electrode exhibits outstanding cycling stability. The initial discharge capacity of FeHCF-P is 131 mAh g^−1^ and remains 109 mAh g^−1^ after 500 cycles, with a capacity retention of 83%. The specific capacity of FeHCF-P decays only 0.0306% per cycle. However, the capacity retention of FeHCF is only 37.6% after 500 cycles. The above results prove that the electrochemical performance of the FeHCF-P electrode can be comprehensively improved after vacancy repairing, which is closely related to the enhancement of the structural stability.

**Fig. 3:**
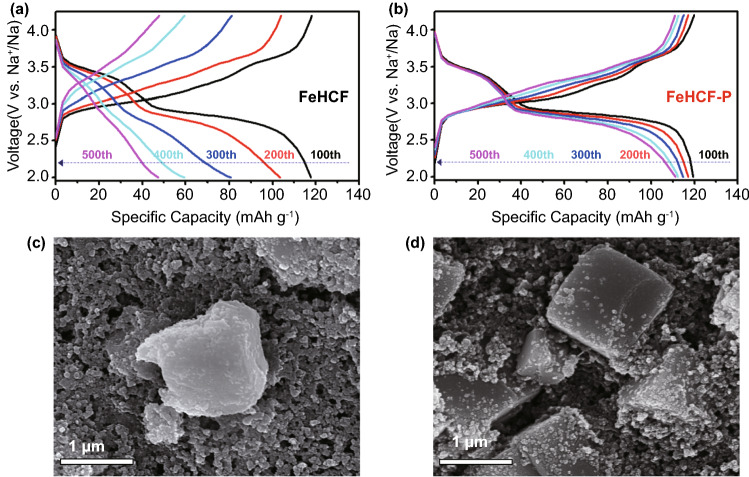
Charge–discharge curves of **a** FeHCF and **b** FeHCF-P at different cycles. SEM images of **c** FeHCF and **d** FeHCF-P electrodes after 300 cycles

To further understand the fading mechanism of FeHCF, the charge–discharge profiles of the FeHCF and FeHCF-P electrodes were compared for different cycles at 1 C (Fig. 3a, b). It shows that both the FeHCF and FeHCF-P electrodes exhibit two prominent discharge platforms at 3.3 and 2.8 V in the 100^th^ cycle. Nevertheless, the discharge capacity and voltage platforms of the FeHCF electrode clearly decay from the 200^th^ cycle (Fig. 3a), and the discharge capacity decreases accordingly, from 103.4 mAh g^−1^ in the 200^th^ cycle to 47.5 mAh g^−1^ in the 500^th^ cycle, where the capacity retention is only 45.9%. Comparatively, the FeHCF-P electrode always exhibits two distinct discharge platforms from the 200^th^ to the 500^th^ cycle (Fig. 3b). Hence, it is speculated that the structure of FeHCF is gradually destroyed during cycling. To prove this speculation, the dissolution behavior of the electrodes in the electrolyte was investigated after 300 cycles at 1C. It is found that the Fe concentration in the electrolyte of the FeHCF-P cell is 0.09 mg L^−1^, which is much lower than that of the FeHCF cell (0.87 mg L^−1^). This result confirms the significant dissolution of FeHCF during cycling, thus leading to a collapse in the structure. The SEM images show that the morphology of FeHCF after 300 cycles is significantly damaged, whereby the surface becomes extremely rough (Fig. 3c). Comparatively, the FeHCF-P electrode maintains the initial cubic particle morphology and its surface remains smooth, proving the high structural stability of FeHCF-P (Fig. 3d). Thus, it is concluded that FeHCF-P prepared by post-synthetic vacancy repairing can effectively inhibit the dissolution of Fe ions and minimize side reactions with electrolytes during the charge–discharge process. This result further proves that the vacancy repairing strategy in a concentrated [Fe(CN)_6_]^4−^ solution can reinforce the crystalline structure of FeHCF and guarantee the cycling stability.

### In-situ Vacancy Repairing Strategy

**Fig. 4:**
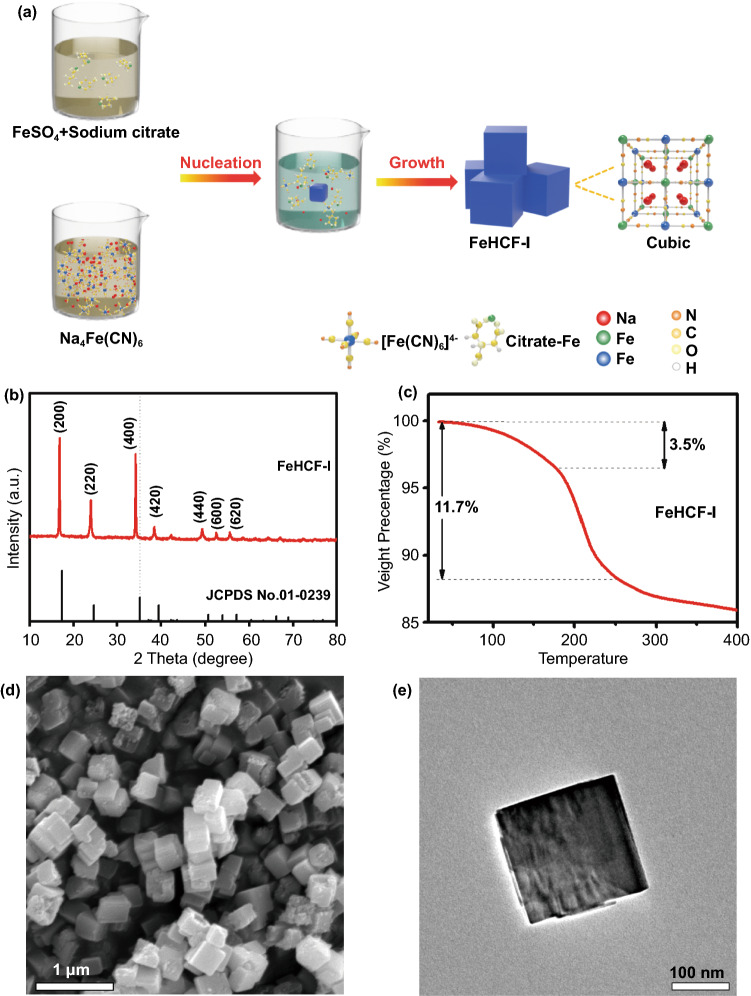
**a** Schematic illustration of the synthetic procedure of FeHCF-I. **b** XRD pattern. **c** TG curve. **d** SEM image and **e** TEM image of FeHCF-I

It should be noted that in the post-synthetic vacancy repairing procedure, the vacancy repairing mainly takes place on the surface, which leads to residual vacancy defects inside the FeHCF particles. To further decrease the vacancy defects, an in-situ vacancy repairing strategy is proposed. High-quality FeHCF-I (in-situ vacancy repaired FeHCF) was synthesized through a co-precipitation method in a saturated Na_4_Fe(CN)_6_ solution. The high content of [Fe(CN)_6_]^4−^ ensured in-situ vacancy repairing during the crystalline growth of FeHCF (Fig. 4a). The XRD pattern shows that a typical face-centered cubic structure is obtained (Fig. 4b). The TGA result shows that the weight loss below 250 ℃ in FeHCF-I is only 11.7%, indicating a low water content (Fig. 4c). Based on elemental analysis data (Table S1), the molecular formula of FeHCF-I was calculated as Na_1.95_Fe[Fe(CN)_6_]_0.88_·1.83H_2_O. The high sodium content is ascribed to the large amount of Na^+^ in the precursor and the low vacancy defects. The XPS spectra show that the chemical valence of the iron element in FeHCF-I mainly remains divalent (Fig. S6). The SEM and transmission electron microscopy (TEM) images reveal a nanocube structure of FeHCF-I with a size distribution of 300 ~ 500 nm. Compared with the microsized FeHCF, the sodium citrate and saturated Na_4_Fe(CN)_6_ in the precursor solution can synergistically inhibit the rapid precipitation and repair the vacancy defects, thus leading to the nanosized particles. Accordingly, the BET surface area of FeHCF-I is 17.10 m^2^ g^−1^, which is larger than that of FeHCF (Fig. S4).

**Fig. 5:**
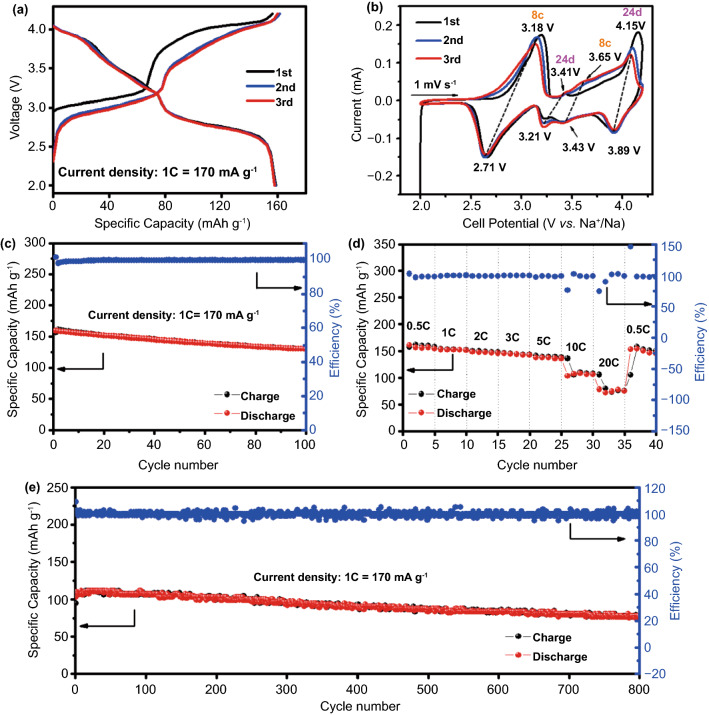
Electrochemical properties of FeHCF-I. **a** Charge–discharge curves at 1 C. **b** CV curves. **c** Cycling performance at 1 C. **d** Rate performance at current densities from 0.5 to 20 C. **e** Cycling stability at 10 C

The electrochemical performance of FeHCF-I is displayed in Fig. 5a. In the first cycle, the FeHCF-I electrode can deliver a high discharge capacity of 158.5 mAh g^−1^ at 1C with an initial coulombic efficiency of 101.4%. Two distinct charging plateaus are observed at 3.15 and 4.1 V, corresponding to the oxidation of HS-Fe and LS-Fe, respectively. Notably, the specific capacity contribution from LS-Fe reaches 80 mAh g^−1^, which is much higher than that of both FeHCF and FeHCF-P (~ 40 mAh g^−1^), implying that fewer vacancy defects exist in FeHCF-I. Considering the average discharge voltage of ~ 3.15 V, a high energy density of 501 Wh kg^−1^ can be achieved for FeHCF-I. Figure 5b shows the CV profiles of the FeHCF-I electrode at 1 mV s^−1^. Four pairs of redox peaks at 3.18/2.71, 3.41/3.21, 3.65/3.43, and 4.15/3.89 V are clearly observed, corresponding to the redox reactions of Fe_HS_-8c, Fe_HS_-24d, Fe_LS_-8c, and Fe_LS_-24d, respectively. The redox pair related to Fe_LS_-24d becomes especially prominent compared with that of FeHCF-P. This phenomenon indicates that the electrochemical activity of LS-Fe is significantly activated, which is consistent with the large capacity contribution from LS-Fe.

The relationship between the LS-Fe capacity contribution and the vacancy defect was further investigated by first-principles density functional theory (DFT) calculations. Table S2 shows the energy state of Na^+^ ions at 8c and 24d sites at different sodium concentrations in a perfect iron hexacyanoferrate framework. It is observed that Na^+^ ions located at 24d sites have the lowest energy level, and the energy decreases with the increasing sodium concentration. This indicates that 24d is the most stable storage site and 8c is the metastable site in iron hexacyanoferrate, which is consistent with previous reports that Na^+^ ions have the priority to occupy 24d sites in a Na-rich FeHCF [20]. However, in a vacancy-rich FeHCF, many 24d sites are destroyed due to the deficiency of [Fe_LS_(CN)_6_]^4−^, resulting in the low capacity contribution from LS-Fe. As for FeHCF-I, in-situ vacancy repairing can effectively reduce the vacancies from the inside-out, thus activating the redox reactions at 24d sites, especially for LS-Fe.

The cycling performance of the FeHCF-I electrode at 1 C is presented in Fig. 5c. The FeHCF-I electrode shows an initial discharge capacity of 158.5 mAh g^−1^, and remains 133.2 mAh g^−1^ after 100 cycles. Figure 5d shows the rate capability of the FeHCF-I electrode at different current densities. The FeHCF-I electrode delivers the initial discharge specific capacity of 160 mAh g^−1^ at 0.5 C. With the increasing current density, FeHCF-I can retain a reversible capacity of 137 mAh g^−1^ at 3 C. Even at a super-high rate of 20 C, the FeHCF-I electrode still maintains a specific capacity of 72 mAh g^−1^. To investigate the long cycling stability, the FeHCF-I electrode was cycled at 10 C (Fig. 5e). The FeHCF-I electrode retains a reversible capacity of 77.8 mAh g^−1^ after 800 cycles, corresponding to a 75% capacity retention of the initial capacity of 103 mAh g^−1^. The excellent rate capability and cycling stability of FeHCF-I originate from its nano-sized and robust framework. Such high specific capacity and rate performance is competitive with other reported hexacyanoferrate cathodes (Fig. S7) [13, 18, 20, 21, 34–37].

**Fig. 6:**
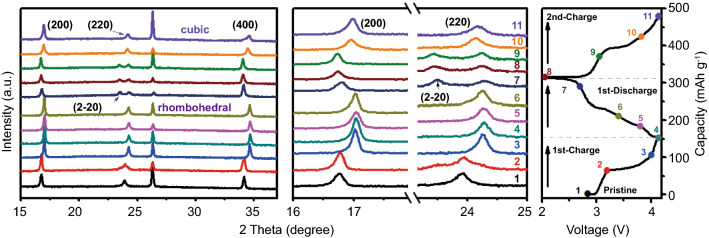
*Ex situ* XRD patterns of FeHCF-I during the charge–discharge process

*Ex situ* X-ray diffraction measurements were employed to investigate the structural reversibility of FeHCF-I during the charge–discharge process (Fig. 6). During the first charge process, the FeHCF-I electrode maintains a cubic phase and the diffraction planes of (200) and (220) gradually shifts to higher angles, indicating the decreasing lattice parameters caused by the continuous extraction of Na^+^. While in the first discharge process, a new diffraction peak appears at 23.5° at 2.7 V, corresponding to the peak splits of the (220) plane to the (220) and (2–20) planes. This also indicates a phase transformation from cubic to rhombohedral. From 2.7 to 2.0 V, the rhombohedral phase remains unchanged, though the diffraction peaks slightly shift toward lower angles. In the following charge process, the rhombohedral phase changes back to the cubic phase. Furthermore, all peaks gradually shift to higher angles, which demonstrates that the FeHCF-I framework is highly reversible and stable due to the in-situ vacancy repairing.

## Conclusion

Based on DFT calculations, post-synthetic and in-situ vacancy repairing strategies were proposed to synthesize high-quality FeHCF in a highly concentrated Na_4_Fe(CN)_6_ solution. The quantity of vacancy defects in FeHCF could be significantly reduced by ~ 26% after post-synthetic vacancy repairing, and the cycling stability was improved accordingly. FeHCF-P delivered a reversible discharge capacity of 131 mAh g^−1^ and remained 109 mAh g^−1^ after 500 cycles, with a capacity retention of 83%. Further investigations showed that FeHCF-P prepared by vacancy repairing could suppress side reactions with electrolytes and inhibit the dissolution of Fe ions during the charge–discharge process. Thus, the vacancy repairing strategy in a concentrated [Fe(CN)_6_]^4−^ solution could effectively reinforce the crystalline structure of FeHCF and guarantee the cycling stability. Furthermore, in-situ vacancy repairing in the solution synthesis of FeHCF could repair the vacancies from the inside-out, demonstrating an enhanced sodium storage performance. FeHCF-I prepared from the in-situ vacancy repairing strategy demonstrated a high discharge capacity of 158.5 mAh g^−1^ at 1 C, with an initial coulombic efficiency of 101.4%. Even at 10 C, the FeHCF-I electrode still maintained a discharge specific capacity of 103 mAh g^−1^ and retained 75% after 800 cycles. *Ex situ* XRD also proved the highly stable framework of FeHCF-I. Thus, in-situ vacancy repairing could reduce the vacancies from the inside-out and activate the redox reactions at 24d sites, especially for LS-Fe. This work provides a new vacancy repairing strategy for the solution synthesis of high-quality FeHCF.

## Supplementary Information

Below is the link to the electronic supplementary material.

Supplementary file1 (PDF 657 kb)
