# Supplementary material for: Post-Synthetic and In Situ Vacancy Repairing of Iron Hexacyanoferrate Toward Highly Stable Cathodes for Sodium-Ion Batteries
Source: Nanomicro Lett. 2021 Dec 3;14:9. doi: 10.1007/s40820-021-00742-z (PMC8642478; doi:10.1007/s40820-021-00742-z)
Supplement: Supplementary file 1 — Supplementary file1 (PDF 657 kb) [file 40820_2021_742_MOESM1_ESM.pdf]

Supporting Information for

Post-Synthetic and In-Situ Vacancy Repairing of Iron Hexacyanoferrate Towards Highly Stable Cathodes for Sodium-Ion Batteries

Min Wan[1], Rui Zeng[2], Jingtao Meng[3], Zexiao Cheng[3], Weilun Chen[3], Jiayu Peng[3], Wuxing Zhang[3, *], Yunhui Huang[3, *]

[1]School of Mechanical Science and Engineering, Huazhong University of Science and Technology, Wuhan 430074, P. R. China

[2]School of Chemistry and Environmental Engineering, Wuhan Polytechnic University, Wuhan 430023, P. R. China

[3]State Key Laboratory of Materials Processing and Die & Mould Technology, School of Materials Science and Engineering, Huazhong University of Science and Technology, Wuhan 430074, P. R. China

*Corresponding authors. E-mail: zhangwx@hust.edu.cn (Wuxing Zhang), huangyh@hust.edu.cn (Yunhui Huang)

Supplementary Table and Figures

Table S1 Elemental contents of FeHCF, FeHCF-P and FeHCF-I (wt%)

	Na	Fe	C	N
FeHCF	12.39	32.79	18.92	21.93
FeHCF-P	11.30	33.26	19.51	22.98
FeHCF-I	14.13	33.31	20.14	23.24

Table S2 Energy levels (eV) of Na^+ at 8c and 24d sites in FeHCF with different sodium concentrations

	Na-0	Na-10	Na-20
8c	-3.26	-4.00	-3.80
24d	-3.44	-4.31	-4.36

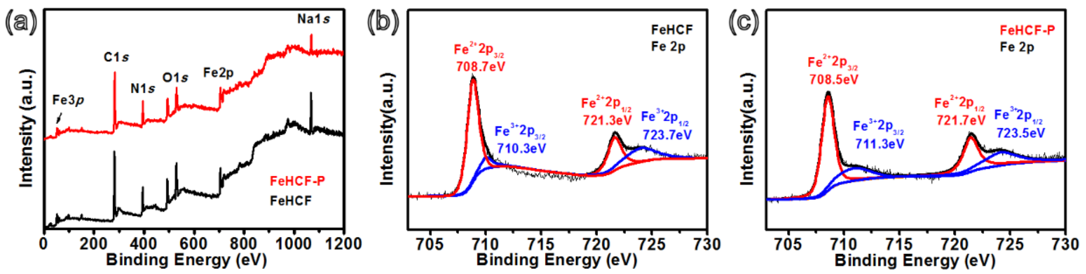

Fig. S1 a XPS survey spectra of FeHCF and FeHCF-P. Fe 2p XPS spectra of **b** FeHCF and **c** FeHCF-P

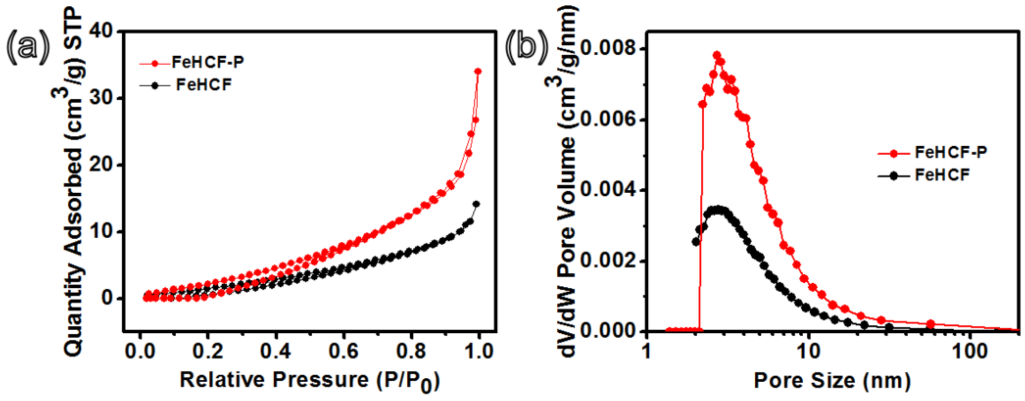

Fig. S2 a N_2 adsorption/desorption isotherm curves and **b** BJH pore-size distribution curves of FeHCF and FeHCf-P

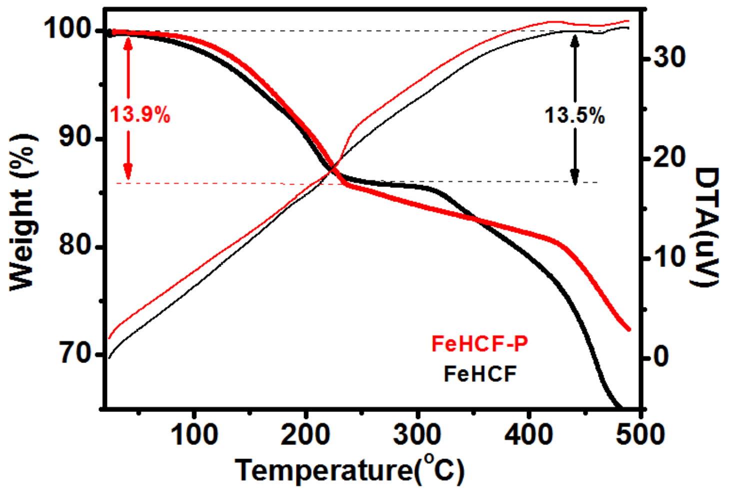

Fig. S3 TG and DTA curves of FeHCF and FeHCF-P in N_2

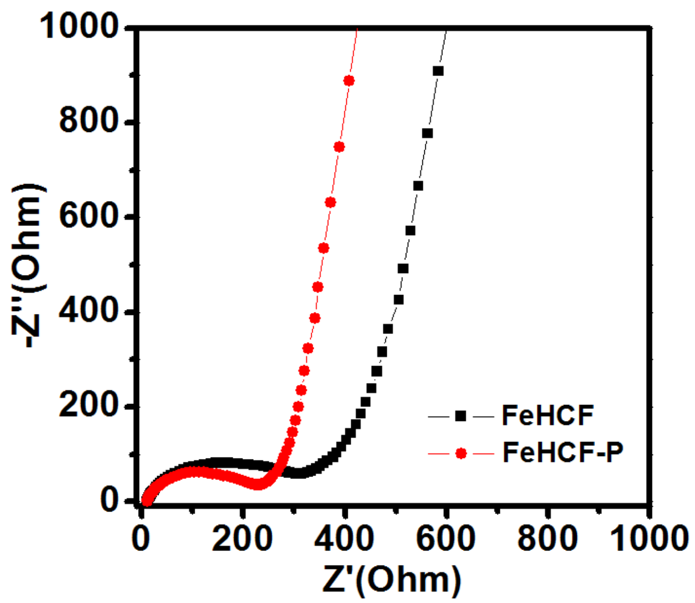

Fig. S4 Electrochemical impedance spectra of FeHCF and FeHCF-P

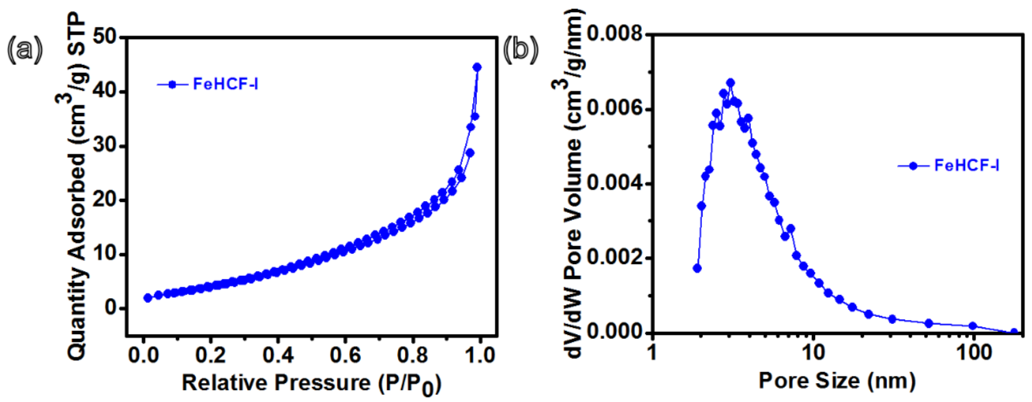

Fig. S5 a N_2 adsorption/desorption isotherm curve and **b** the BJH pore-size distribution curve for FeHCF-I

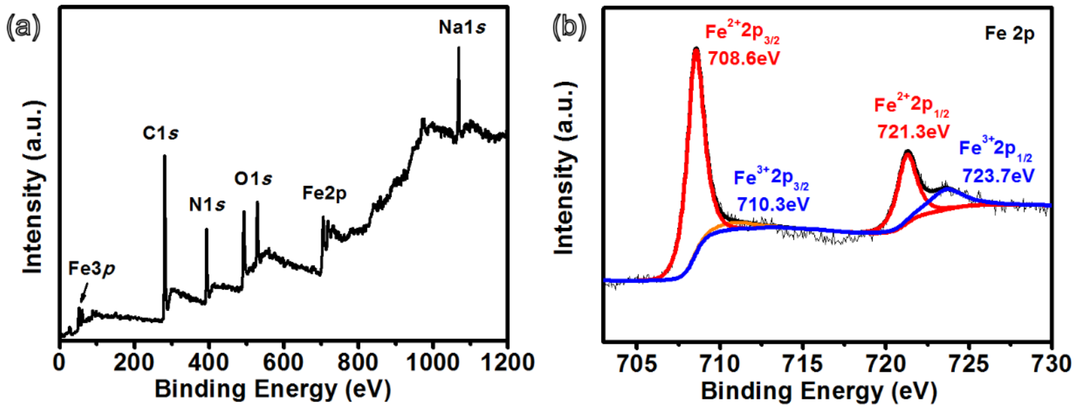

Fig. S6 a XPS survey spectrum and **b** Fe 2p XPS spectrum of FeHCF-I

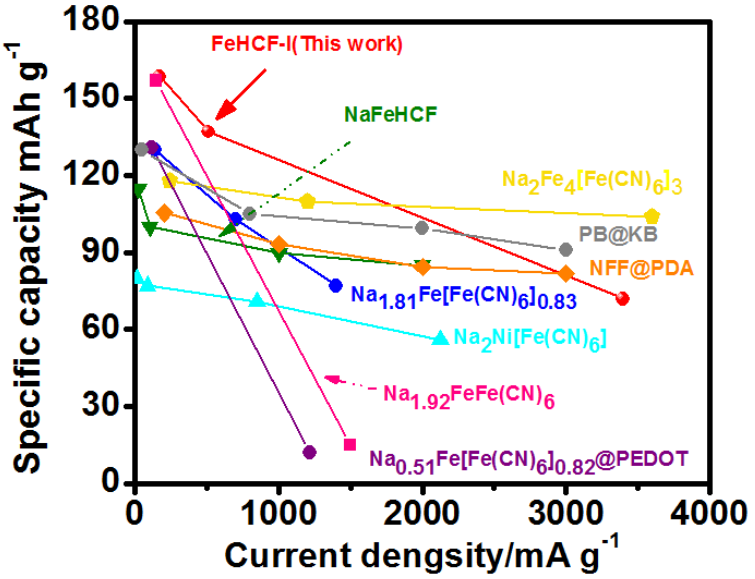

Fig. S7 Comparison of rate performance between FeHCF-I and reported PBAs cathode in previous literatures
